# How can clinicians, specialty societies and others evaluate and improve the quality of apps for patient use?

**DOI:** 10.1186/s12916-018-1211-7

**Published:** 2018-12-03

**Authors:** Jeremy C. Wyatt

**Affiliations:** Wessex Institute of Health, Faculty of Medicine, University of Southampton, Southampton, SO16 7NS UK

**Keywords:** Digital healthcare, mHealth, Health apps, Smart phone, Mobile phone, Quality and safety, Evaluation methods, Quality checklist, Regulation, e-Health, Health policy

## Abstract

**Background:**

Health-related apps have great potential to enhance health and prevent disease globally, but their quality currently varies too much for clinicians to feel confident about recommending them to patients. The major quality concerns are dubious app content, loss of privacy associated with widespread sharing of the patient data they capture, inaccurate advice or risk estimates and the paucity of impact studies. This may explain why current evidence about app use by people with health-related conditions is scanty and inconsistent.

**Main text:**

There are many concerns about health-related apps designed for use by patients, such as poor regulation and implicit trust in technology. However, there are several actions that various stakeholders, including users, developers, health professionals and app distributors, can take to tackle these concerns and thus improve app quality. This article focuses on the use of checklists that can be applied to apps, novel evaluation methods and suggestions for how clinical specialty organisations can develop a low-cost curated app repository with explicit risk and quality criteria.

**Conclusions:**

Clinicians and professional societies must act now to ensure they are using good quality apps, support patients in choosing between available apps and improve the quality of apps under development. Funders must also invest in research to answer important questions about apps, such as how clinicians and patients decide which apps to use and which app factors are associated with effectiveness.

**Electronic supplementary material:**

The online version of this article (10.1186/s12916-018-1211-7) contains supplementary material, which is available to authorized users.

## Background

Apps are interactive software tools designed to run on mobile phones, tablet computers or wearable devices, which use data entered by the user, from sensors or other sources, to deliver a huge variety of functions to the user, tailored to their needs. There is considerable concern among health care professionals about the quality of apps for patient or professional use [1–3], how patients use apps and whether they reveal this use in a consultation. Some clinicians worry that, while using apps, patients may incur risks that could rival those associated with complementary therapies. Another concern is how clinicians should use the patient data collected by apps, which may be captured more frequently than in the clinic but will rarely use a calibrated measurement device or validated questionnaire. Apart from these measurement issues, it is often unclear to clinicians whether the variability of frequently measured data items recorded by apps, such as blood glucose levels or heart rate, reflects normal or “special cause” variation [4].

This article aims to help clinicians (and their patients) to avoid the worst quality, unsafe apps and to provide a framework for assessing and distinguishing between apps that may seem acceptable at first glance. I review the importance of apps, how patients use them, the quality issues surrounding apps and their use by clinicians and patients and why they arise. Then, I discuss existing methods to assure the quality and assess the risk of different apps, describe methods to evaluate apps and provide advice to clinicians about the kinds of app that may be recommended and to which patients. Finally, I describe how clinicians acting together as members of a specialty society can contribute to a curated generic app repository, listing priority actions and suggested research questions.

The apps under consideration here are those which aim to educate, motivate or support patients about their symptoms, diagnosed illness or the therapies or monitoring required to keep diseases in check. Some patient apps are also intended to be therapeutic; for example, by delivering interactive cognitive behaviour therapy (see Box 1).

### Why are patient apps important?

Cash-strapped health systems are simultaneously encountering increasing numbers of elderly patients with multiple conditions, while facing staff recruitment challenges. So, many organisations encourage patient self-management and see apps and mHealth (the use of mobile phones and wearables as tools to support healthcare delivery and self-care) as a panacea to support this [5]. Good evidence of app effectiveness is lacking in most disease areas [3]. However, it is largely agreed that apps have great potential to support self-management and improve patients’ experiences and outcomes of disease, particularly considering that, throughout their waking hours, most adults and teenagers carry a mobile phone with a camera and high resolution screen able to deliver reminders and capture data from wearable technology and other devices via Bluetooth. Smart phones also have multiple sensors, allow communication in several ways (speech, text, video – even virtual reality) and run apps, which – because they usually deliver a tailored experience – are more likely to improve the effectiveness of behaviour change [6]. Apps thus provide health systems and clinicians around the world with an alternative to direct care, reaching very large numbers of patients at marginal cost. The fact that apps are scalable, while face-to-face encounters are not, helps explain the high expectations of app developers, health systems and service managers.

### Evidence about the usage of apps by patients

Unfortunately, so far, we know rather little about how patients use apps. One study [7] of 189 diabetics attending a New Zealand outpatient clinic (35% response rate) found that 20% had used a diabetes app, younger people with type 1 diabetes were more likely to use apps, and a glucose diary (87%) and insulin calculator (46%) were the most desirable features. A glucose diary was also the most favoured feature in non-users (64%) [7]. Another recent survey [8] of 176 people with depression or anxiety seeking entry to a US trial of mental health apps (not a representative sample of all people with mental health issues) showed that 78% claimed to have a health app on their device, mainly for exercise (53%) or diet (37%). Only 26% had a mental health or wellness app on their device. The mean number of health apps on each person’s device was 2.2, but the distribution was heavily skewed (SD 3.2). Two-thirds of respondents reported using health apps at least daily [8].

## What are the issues with apps and how do these arise?

**Fig. 1:**
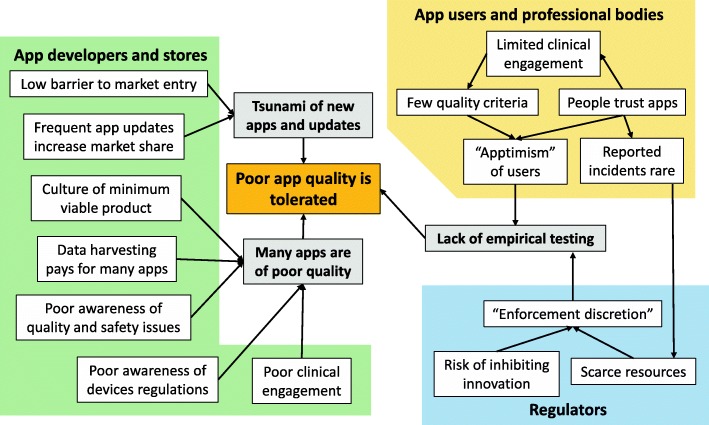
Reasons why poor app quality is common and widely tolerated. These include the large number of apps, poor clinical engagement and understanding by developers, and lack of empirical testing

There are several reasons why apps are not yet an ideal route for delivering high quality, evidence-based support to patients (see Fig. 1).

### The role of app developers and distributors

Nowadays, anyone can develop an app using, for example, the MIT App Inventor toolkit [9]; in fact, 24 million apps have been developed using this toolkit since 2011. This low barrier to entering the app marketplace means that most medical app developers come from outside the health field. They may fail to engage sufficiently with clinicians or patients [10], or to consider safety or effectiveness, because they are unaware of the regulations surrounding medical devices and existing app quality criteria [11]. The entrepreneurial model means that many incomplete apps are rushed to market as a ‘minimum viable product’ [12], with the intention to incrementally improve them based on user feedback. Often, however, this does not happen [10]. As a result, many apps are immature and not based on evidence, so are not clinically effective [13].

Many health apps are free, paid for by the harvesting of personal data for targeted marketing [14] – an industry worth $42 billion per year [15]. This means that personal – often sensitive – data are being captured and transmitted in an identifiable, unencrypted form [16] across the globe. While Apple restricts the types of app that developers can upload to its App Store (see below), other app distributors have much looser requirements, with many free apps being thinly disguised vehicles for hidden trackers and user surveillance [14]. Thus, many of the patient apps on these other app repositories are of poor quality [17], while some are frankly dangerous. For example, in a study of the performance of melanoma screening apps, four out of five were so poor that they could pose a public health hazard by falsely reassuring users about a suspicious mole. This might cause users to delay seeking medical advice until metastasis had occurred [18]. The only accurate app worked by taking a digital photograph of the pigmented lesion and sending it to a board-certified dermatologist.

### The role of app users, health professionals and regulators

Unfortunately, patients and health professionals are also partly to blame for the problems of inaccuracy, privacy erosion and poor app quality. Most of us carry and use our smartphone all day, so we trust everything it brings us. This leads to an uncritical, implicit trust in apps: ‘apptimism’ [19]. This is exacerbated by the current lack of clinical engagement in app development and rigorous testing and poor awareness of app quality criteria. Low rates of reporting faulty apps or clinical incidents associated with app use mean that regulators cannot allocate sufficient resources to app assessment. The large numbers of new health apps appearing (about 33 per day on the Apple app platform alone [20]) and government support for digital innovation means that some regulators adopt a position of ‘enforcement discretion’ [21]; i.e., they will not act until a serious problem emerges. Apptimism and ‘digital exceptionalism’ [22] also mean that the kind of rigorous empirical studies we see of other kinds of health technologies are rare in the world of apps. The result is that most health-related apps are of poor quality (see Table 1), but this situation is widely tolerated [23].

**Table 1:** Some of the quality issues associated with health-related apps

Problem area	Examples
Privacy issues	Lengthy privacy policies, harvesting of personal data with identifiers, transmission of sensitive data unencrypted [16]
Poor quality content	Acne treatment apps using iPhone screen radiation [43] No evidence underlying apps for smoking cessation [13]
Vague or misleading description of app purpose	Breath alcohol detection app for phone with no alcohol sensor [44]
Poor app usability	“Usability problem ratings ranged from moderate to catastrophic” in apps for type 2 diabetes [17]
Ranking and cost not correlated with content	Shown for smoking cessation apps [13]
Variable accuracy	Melanoma apps [18] Opiate dose calculator [39] Cardiovascular risk calculator accuracy [45] Heart rate apps [46]

## How we can improve app quality and distinguish good apps from poor apps?

### Summary of existing methods to improve app quality

Several strategies can be used by various stakeholders to help improve the quality of an app at each stage in its lifecycle, from app development to app store upload, app rating, its use for clinical purposes and finally its withdrawal from the app distributor’s repository when it is no longer available or of clinical value (Table 2). Apple has already put some of the strategies into action [24] (see Box 2).

**Table 2:** Potential stakeholders and roles in improving app quality along the app lifecycle

Stage in app lifecycle	Stakeholders	Potential quality improvement processes	Examples of these processes
1. Development	Developers, users, clinicians, standards bodies	Involve clinicians and users Refer to engineering standards Understand app quality criteria Develop and evaluate app using appropriate framework	BSI app standard PAS 277 [47] RCP checklist [19] MRC digital intervention development process [38]
2. Uploading to app repository	App repository owners	Check technical aspects Check privacy Check developer qualifications Check CE mark	Apple App Store excludes drug-related apps unless developer is a product licence holder (see Box 2)
3. App rating and review	Raters	Wisdom of the crowd Use explicit criteria	Can fail [13] RCP checklist [19]
4. Selection from the app repository	App repository owners	Check quality Check CE mark, intended app user, training needed, etc.	Complete app risk checklist [27] Check if needs CE mark
	Users	Consider risks Check reviews	Read iMedicalApps review Select from a curated app repository Seek doctor’s advice RCP checklist [19]
5. Using app for self-management	Users	Use with caution Notify doctor or regulator of errors or near-misses	RCP guidance for physicians [19]
6. Removal from app repository	Regulators, app repository owners	Respond to reviews, reports of adverse events, lack of evidence to support claims	Apple’s stance on health related apps (see Box 2) Banning of some ineffective acne apps [43]

Abbreviations: *BSI* British Standards Institution, *CE* Conformité Européene, *MRC* Medical Research Council, *PAS* Publically available specification, *RCP* Royal College of Physicians

**Fig. 2:**
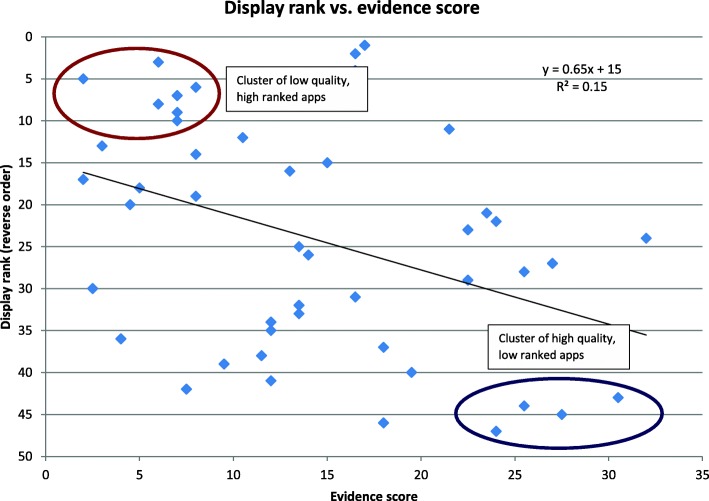
Comparison of Apple iTunes App Store or Google Play store rank (vertical axis, inverse scale) with the quality of the underlying evidence on which 47 smoking cessation apps are based. The higher the evidence score (x axis), the more the app conforms to relevant guidelines from the US Preventive Service Task Force. The lower the store rank (y axis, reverse scale), the higher the app is listed in the App Store or Google Play store. The brown ellipse shows a cluster of low quality, high ranked apps, while the blue ellipse shows a cluster of high quality, low ranked apps. Author’s analysis based on data from Abroms et al. [13]

Unfortunately, poor quality apps still rise to the top of the list in various app repositories. Figure 2 compares the ranking of 47 smoking cessation apps from Apple and Android app stores with the quality of their knowledge base (author re-analysis based on data from [13]). While the apps are widely scattered along both axes, there is a negative correlation of quality with ranking, suggesting a broken market.

### App checklists

One approach to improve quality is checklists for app users, or for physicians recommending apps to patients. Several checklists exist [25, 26], but few have professional support for their content. One exception is the UK Royal College of Physicians (RCP) Health Informatics Unit checklist of 18 questions [19] exploring the structure, functions and impact of health-related apps (see Additional file 1 for details).

### Assessing the risks associated with health app use

To help regulators and others to focus on the few high-risk apps hidden in the deluge of new apps, Lewis et al. [27] described how app risk is associated with app complexity and functions. They point out that risk is related to the context of app use [27], including the user’s knowledge and the clinical setting. Paradoxically, this risk may be higher in community settings rather than in clinical settings such as intensive care units, where patients are constantly monitored and a crash team is on hand. Contrast this with an elderly diabetic who is only visited at weekends, who uses an app to adjust her insulin dose levels at home [27].

## How can we evaluate apps?

### A common-sense app evaluation framework

The next stage is to test the accuracy of any advice or risks calculated. The methods are well established for decision support systems [28], predictive models [29] and more generally [30]. To summarise, investigators need to:

1. Define the exact question; for example, “how accurately does the app predict stroke risk in people with cardiovascular disease aged 60–85?”
2. Assemble a sufficiently large, representative test set of patients who meet the inclusion criteria, including the ‘gold standard’ for each. This gold standard can be based on follow-up data or on expert consensus for questions about the appropriateness of advice, using the Delphi technique.
3. Enter the data (ideally, recruit typical app users for this), recording the app’s output and any problems; for example, cases in which the app is unable to produce an answer.
4. Compare the app’s results against the gold standard using two-by-two tables, receiver operating characteristic (ROC) curves and a calibration curve to measure the accuracy of any probability statements. For details of these methods, see Friedman and Wyatt [30].

Assuming accurate results in laboratory tests, the next question is: “does the app influence users’ decisions in a helpful way?” This is important because poor wording of advice or presentation of risk, inconsistent data entry, or variable results when used offline may reduce its utility in practice. To answer this question, we can use the same test data but instead examine how the app’s output influences simulated decisions in a within-participant before/after experiment [31]. Here, members of a group of typical users review each scenario and record their decisions without the app, then enter the scenario data into the app and record their decision after consulting it [30, 31]. This low cost study design is faster than a randomised clinical trial (RCT) and estimates the likely impact of the app on users’ decisions if they use it routinely. It also allows us to estimate the size of any ‘automation bias’; i.e., the increase in error rate caused by users mistakenly following incorrect app advice when they would have made the correct decision without it [32, 33].

The most rigorous app evaluation is an RCT of the app’s impact on real (as opposed to simulated) user decisions and on the health problem it is intended to alleviate [28, 34]. Some app developers complain that they lack the funds or that their software changes too frequently to allow an RCT to be conducted. However, at least 57 app RCTs have been conducted [35] and there are variant RCT designs that may be more efficient.

### New methods to evaluate apps

The *Interactive Mobile App Review Toolkit* (IMART) [36] proposes professional, structured reviews of apps that are stored in a discoverable, indexed form in a review library. However, this will require a sufficient number of app reviewers to follow the suggested structure and to keep their reviews up to date, while app users need to gain sufficient benefit from consulting the library to make them return regularly. Time will tell whether or not these requirements are met.

While expert reviews will satisfy some clinicians, many will wait for the results of more rigorous studies. Variants on the standard RCT, including cluster trials, factorial trials, step-wedge designs or multiphase optimisation followed by sequential multiple assignment trials (MOST-SMART) [37] may prove more appropriate. These methods are summarised in a paper on the development and evaluation of digital interventions from an international workshop sponsored by the UK Medical Research Council (MRC), US National Institutes of Health (NIH) and the Robert Wood Johnson Foundation [38].

## Advice to clinicians who recommend apps to patients

There are several ways in which physicians can improve the quality of apps used by patients, including:

1. Working with app developers to identify measures that would improve the quality of their app, contributing directly to the development process by, for example, identifying appropriate evidence or a risk calculation algorithm on which the app should be based
2. Carrying out and disseminating well-designed evaluations of app accuracy, simulated impact or effectiveness, as outlined above
3. Reporting any app that appears to threaten patient safety or privacy to the appropriate professional or regulatory authority, together with evidence
4. Using a checklist – such as that reproduced above – to carry out an informal study of apps intended for use by patients with certain conditions; communicating the results of this study to individual patients or patient groups; regularly reviewing these apps when substantial changes are made
5. Raising awareness among peer and patient groups of good quality apps, those that pose risks, the problem of ‘apptimism’, the app regulatory process and methods to report poor quality apps to regulators
6. Working with professional societies, patient groups, regulators, industry bodies, the media or standards bodies to promote better quality apps and public awareness of this.

### What kinds of app should a physician recommend?

Apps often include several functions and it is hard to give firm advice about which functions make clinical apps safe or effective. For example, we do not yet know which generic app features – such as incorporating gaming, reminders, tailoring or multimedia – are associated with long term user engagement and clinical benefit. Instead, the clinician is advised to check each app for several features that most workers agree suggest good quality (see Box 3). They should then satisfy themselves that the app functions in an appropriate way with some plausible input data, in a scaled-down version of the full accuracy study outlined earlier.

However, even a high quality app can cause harm if it is used by the wrong kind of patient, in the wrong context or for the wrong kind of task.

### To which kinds of patients and in what context?

Apps are most effective when used by patients with few sensory or cognitive impairments and stable, mild-to-moderate disease, in a supervised context. In general, we should probably avoid recommending apps to patients with unstable disease or to those who are frail or sensory impaired, especially to patients in isolated settings where any problem resulting from app misuse, or use of a faulty app, will not be detected quickly. Clinicians need to think carefully before recommending apps to patients with certain conditions that tend to occur in the elderly (such as falls, osteomalacia or stroke) or illnesses such as late stage diabetes that can cause sensory impairment. We do not yet know how user features such as age, gender, educational achievement, household income, multiple morbidity, or health and digital literacy interact with app features, or how these user features influence app acceptance, ease of use, long term engagement and effectiveness. Further research is needed to clarify this.

### For which health-related purposes or tasks?

Many apps claim to advise patients about drug doses or risks. However, even apps intended to help clinicians calculate drug doses have been found to give misleading results (e.g. opiate calculators [39]). As a result, in general, clinicians should avoid recommending apps for dosage adjustment or risk assessment unless they have personally checked the app’s accuracy, or read a published independent evaluation of accuracy.

By contrast, apps for lower risk tasks, such as personal record keeping, preventive care activities (e.g. step counters) or generating self-care advice, are less likely to cause harm. This remains largely true even if the app is poorly programmed or based on inappropriate or out-dated guidance, although it may lead patients to believe that they are healthier than they really are. One exception, however, is where, by following advice from an app, a patient with a serious condition might come to harm simply by delaying contact with a clinician – as with the melanoma apps mentioned earlier [18].

## The role of professional and healthcare organisations in improving access to high quality apps

The world of apps is complex and changes quickly, so while clinicians can act now to help patients choose better apps and work with developers to improve the quality of apps in their specialty, in the longer term it is preferable for professional societies or healthcare organisations to take responsibility for app quality. Indeed, some organisations have already started to do this (e.g. NHS Digital and IQVIA).

**Fig. 3:**
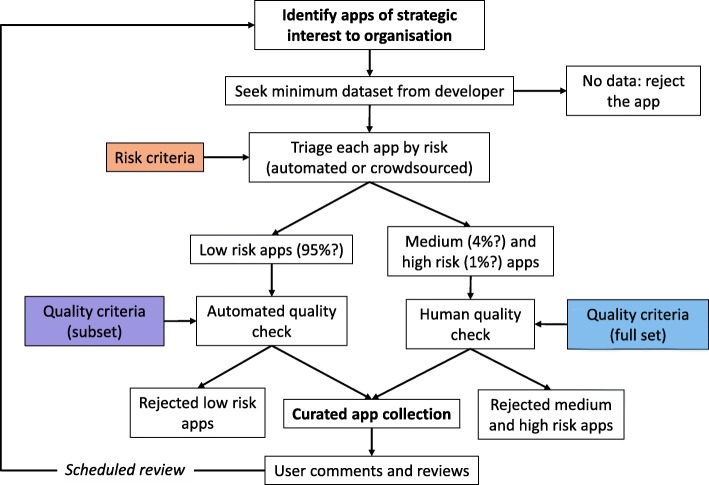
Suggested process for organisations to establish a sustainable curated app repository, based on explicit quality and risk criteria

One method that such organisations can follow is to set up a ‘curated’ app repository that includes only those apps meeting minimum quality standards. Figure 3 suggests how organisations might establish such an app repository, minimising the need for human input. Organisations should first identify the subset of apps of specific interest to them, then capture a minimum dataset from app developers to enable them to carry out a risk-based app triage. Any developer who does not provide the requested data rules their app out at this stage by not acting collaboratively. To minimise demands on professional time, app triage can be automated or crowdsourced by patients with the target condition. Apps that appear low risk are subjected to automated quality assessment, with those that pass being rapidly added to the curated app repository. To minimise the need for scarce human resources, the threshold for judging apps to be of medium and high risk should be set quite high, so they form only a small proportion of the total (e.g. 4% and 1%, respectively). This is because these apps will go through a more intensive, slower manual process, using extended quality criteria before being added to the app repository or being rejected. Importantly, all users of all grades of apps are encouraged to submit structured reviews and comments, which can then influence the app’s place in the app repository.

## Actions to be taken by various stakeholders

Some suggested priority actions for clinicians and professional societies are:

1. To confirm that any apps they use that support diagnosis, prevention, monitoring, prediction, prognosis, treatment or alleviation of disease carry the necessary CE mark. If the mark is missing, the clinician should discontinue use and notify the app developer and the regulator of this, e.g. for the Medicines and Healthcare Products Regulatory Agency (MHRA): devices.implementation@mhra.gov.uk
2. To review the source, content and performance of other apps to check that they meet basic quality criteria
3. To develop an initial list of apps that seem of sufficient quality to recommend to colleagues, juniors and patients
4. To report any adverse incidents or near-misses associated with app use to the app developer and the relevant regulator
5. To develop specialty-specific app quality and risk criteria and then begin to establish a curated community app repository
6. To consider collaborating with app developers to help them move towards higher standards of app content, usability and performance, as well as clinically relevant, rigorous evaluations of safety and impact

However, there are other stakeholders and possible actions, some of which are already in progress. For example, the 2017 EU Medical Device Regulation will require more app developers to pay a ‘notified body’ to assess whether their app meets ‘essential requirements’ (e.g., “software that are devices in themselves shall be designed to ensure repeatability, reliability and performance in line with their intended use”). It will also make app repositories the legal importer, distributor or authorised representative and thus responsible for checking that apps carry a CE mark and Unique Device Identifier where required, and responsible for recording complaints and passing them back to the app developer. This Regulation applies now and will become the only legal basis for supplying apps across the EU from May 2020 [40].

## Conclusions

Apps are a new technology emerging from babyhood into infancy, so it is hardly surprising to see teething problems and toddler tantrums. The approach outlined above – understanding where the problems originate and possible actions stakeholders can take, then suggesting ways in which doctors can constructively engage – should help alleviate some current quality problems and ‘apptimism’. The suggestions made here will also help clinicians to decide which apps to recommend, to which patients and for which purposes. Establishing a sustainable, curated app repository based on explicit risk and quality criteria is one way that professional societies and healthcare organisations can help.

This overview raises several research questions around apps and their quality, of which the following seem important to investigate soon:

1. How do members of the public, patients and health professionals choose health apps and which quality criteria do they consider important?
2. Which developer and app features accurately predict acceptability, accuracy, safety and clinical benefit in empirical studies?
3. What is the clinical and cost effectiveness of apps designed to support self-management in common acute or long term conditions?
4. Which generic app features (such as incorporating gaming, reminders, tailoring or multimedia) are associated with long-term user engagement and clinical benefit?
5. How does app acceptance, ease of use, long term engagement and effectiveness vary with user features such as age, gender, educational achievement, household income, multiple morbidity, frailty or health and digital literacy?
6. What additional non-digital actions, such as general practitioner recommendations or peer support, improve user engagement with, and the effectiveness of, self-management apps?

Answering these questions should help apps to pass smoothly from childhood into adulthood and deliver on their great potential – though some unpredictable teenage turmoil may yet await us.

## Box 1. Functions of apps intended for use by patients (many apps include several functions [27])

1. Diagnostic or triage tools to help people understand their symptoms and navigate their way around the health system

2. Education about an illness, its risk factors and how to reduce them, and disease management

3. Tools such as games designed to motivate the patient to self-monitor, learn more about their illness, or adhere to therapy or appointments

4. Reminders to take medications, record observations or attend appointments

5. Record keeping or record access tools, e.g. a mood monitor, log for blood sugar or peak flow readings, or tools to access a personal or official health record and interpret or comment on record entries

6. Risk assessment or disease activity monitoring, e.g. a tool to identify neutropaenic sepsis in patients following chemotherapy based on symptoms, temperature or home-based tests

7. Tools that deliver interactive therapy, e.g. cognitive behaviour therapy or mindfulness training

## Box 2. Statements by Apple about how it ensures the quality of health-related apps [24]

"If your app behaves in a way that risks physical harm, we may reject it. For example:

1.4.1 Medical apps that could provide inaccurate data or information, or that could be used for diagnosing or treating patients may be reviewed with greater scrutiny.

- Apps must clearly disclose data and methodology to support accuracy claims relating to health measurements, and if the level of accuracy or methodology cannot be validated, we will reject your app. For example, apps that claim to take x-rays, measure blood pressure, body temperature, blood glucose levels, or blood oxygen levels using only the sensors on the device are not permitted.
- Apps should remind users to check with a doctor in addition to using the app and before making medical decisions.

If your medical app has received regulatory clearance, please submit a link to that documentation with your app.

1.4.2 Drug dosage calculators must come from the drug manufacturer, a hospital, university, health insurance company, or other approved entity, or receive approval by the FDA or one of its international counterparts. Given the potential harm to patients, we need to be sure that the app will be supported and updated over the long term."

## Box 3. Features that suggest an app is of good quality

The app:

1. Carries a CE (Conformité Européene) mark (although a CE mark does not guarantee quality [41])
2. Is produced or endorsed by an organisation with a reputation to lose, e.g. a professional body, specialty society or medical publisher; or a patient, healthcare or academic organisation
3. Describes the source of knowledge or algorithms used; this source is appropriate and up to date
4. Describes the purpose of the app, the target user and their assumed skills
5. Keeps up with smartphone software updates and with new medical knowledge
6. Has a professional look and feel with clear wording for questions or advice and easy navigation though screens and menus
7. Has an output that appears helpful and appropriate, given sample input data
8. Requests no identifying information, or the information collected is proportionate to the app’s purpose and is accompanied by a brief, easily understood privacy policy. This policy states that no personal data acquired though the app are stored on the mobile device and any health-related data are encrypted before transmission to remote servers. The F-Droid app store carries many examples of such apps [14].

The app developer:

1. Appears on a list of accredited clinical software developers, based on their past products [42]
2. Has followed a structured framework when developing the app, e.g. the MRC framework for complex interventions or variations to this framework proposed by Murray [38]
3. Provides a simple means for users to feed back comments or issues and there is evidence of the developer responding to these
4. Anticipates potential clinical risks that could arise when using the app (e.g. by minors or by those unable to give informed consent) and addresses these using relevant design features
5. Links to independent evidence that the app meets the manufacturer’s claims, either in the form of a published article or an authoritative, independent, attributable review

## Additional file

Additional file 1: RCP Health Informatics Unit clinical app quality checklist: A checklist devised by the Royal College of Physicians’ Health Informatics Unit to help clinicians determine the quality of health-related apps. (DOCX 20 kb)

## Abbreviations

BSI: British Standards Institution
CE: Conformité Européene
MHRA: Medicines and Healthcare Products Regulatory Agency
IMART: Interactive Mobile App Review Toolkit
MOST-SMART: Multiphase optimisation followed by sequential multiple assignment trials
MRC: Medical Research Council
NIH: National Institutes of Health
PAS: Publically available specification
RCP: Royal College of Physicians
RCT: Randomised controlled trial
