# Correction to: How can clinicians, specialty societies and others evaluate and improve the quality of apps for patient use?

**DOI:** 10.1186/s12916-019-1381-y

**Published:** 2019-07-20

**Authors:** Jeremy C. Wyatt

**Affiliations:** Wessex Institute of Health, Faculty of Medicine, University of Southampton, Southampton, SO16 7NS UK

**Correction to: BMC Med**

**https://doi.org/10.1186/s12916-018-1211-7**

Since the publication of this article [1] it has come to my attention that it contains an error in which the y-axis in Fig. 2 was inverted, thus incorrectly displaying a weak negative correlation rather than a weak positive one. This error was introduced as the order of the data on which Fig. 2 was based [2] was misread. The corrected version of Fig. 2 can be seen below, in which a weak positive correlation is now displayed. This does not change the general point, that app users and app stores appear to take little notice of the source of information on which apps are based. I apologise to readers for this error.

**Fig. 2:**
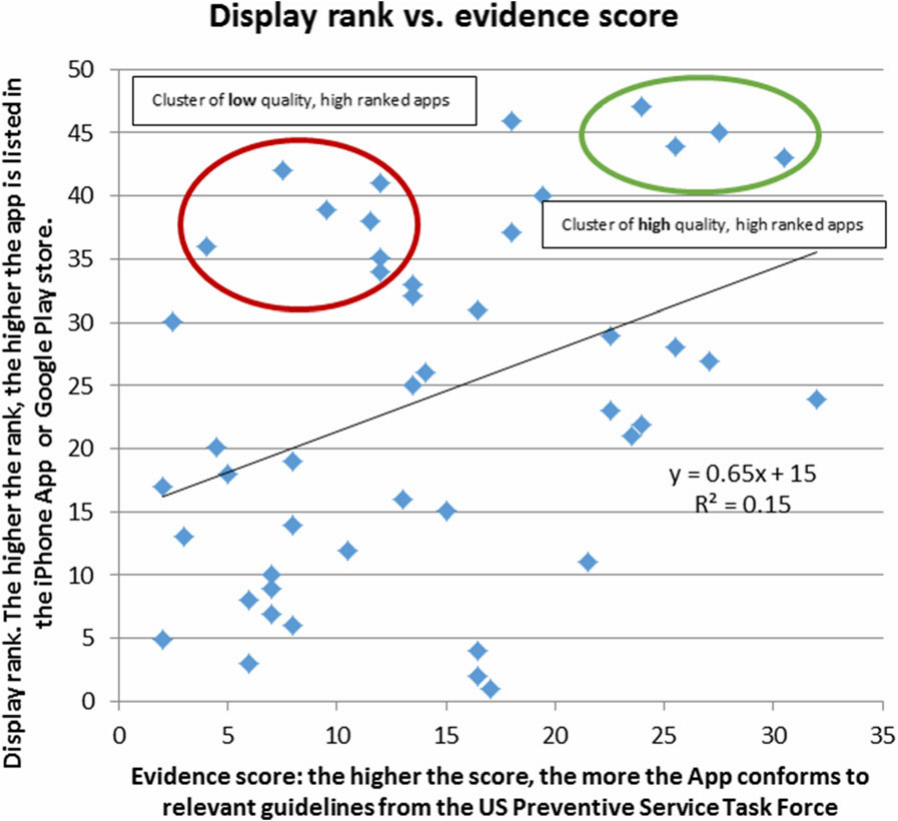
Comparison of Apple iTunes App Store or Google Play store rank (vertical axis, inverse scale) with the quality of the underlying evidence on which 47 smoking cessation apps are based. The higher the evidence score (x axis), the more the app conforms to relevant guidelines from the US Preventive Service Task Force. The lower the store rank (y axis, reverse scale), the higher the app is listed in the App Store or Google Play store. The brown ellipse shows a cluster of low quality, high ranked apps, while the blue ellipse shows a cluster of high quality, low ranked apps. Author’s analysis based on data from Abroms et al. [13]
