# Supplementary material for: How can clinicians, specialty societies and others evaluate and improve the quality of apps for patient use?
Source: BMC Med. 2018 Dec 3;16:225. doi: 10.1186/s12916-018-1211-7 (PMC6276222; doi:10.1186/s12916-018-1211-7)
Supplement: Supplementary file 1 — RCP Health Informatics Unit clinical app quality checklist: A checklist devised by the Royal College of Physicians’ Health Informatics Unit to help clinicians determine the quality of health-related apps. (DOCX 20 kb) [file 12916_2018_1211_MOESM1_ESM.docx]

**Appendix. RCP Health Informatics Unit clinical app quality checklist [20]**

1. **Who developed the app and what’s inside it?**
2. Is it clear who this app is for, and how it should be used? Yes/No/Don’t know
3. Is it clear which problem the app is designed to alleviate or what outcome it helps to promote? Yes/No/Don’t know
4. Does the app developer/sponsor seem well informed about this problem or outcome and likely to be unbiased in their approach to it? Yes/No/Don’t know
5. Have they located sound, relevant, up-to-date evidence, images, video etc. to include in their app? Yes/No/Don’t know
6. Do the app screens look well-designed, is text clear? Not applicable/Yes/No/Don’t know
7. Is it clear which data the app needs from the user, with units defined, out of range detection and a ‘Clear last patient’ button? Not applicable/Yes/No
8. Does the app collect any identifiable patient information? Yes/No/Unclear
9. Does it seem to keep user and patient data secure and private? Yes/No/Don’t know
10. If the app is designed to support a medical task, is it CE marked? Yes/No/Unclear
11. **How well does the app work?**
12. Is the app fast and easy to use in clinical settings? Yes/No/Don’t know
13. Does the app give the user usable answers or advice, quickly? Not applicable/Yes/No/Unclear
14. Do the answers, advice or calculated risks appear to be correct? Yes/No/Unclear
15. Is there a way to feed back user comments to the app developer? Yes/No/Don’t know
16. **Is there any evidence that the app alleviates the problem?**
17. Have any studies been carried out to measure the impact of using the app on clinical or patient knowledge, actions or (preferably) patient outcomes? Yes/No/Don’t know
18. Were these studies independently conducted, well designed, large enough and applicable to the user? Not applicable/Yes/No/Don’t know
19. Did any study also examine health resource use, potential harms caused by the app, or quantify cost effectiveness? Not applicable/Yes/No/Don’t know
20. Overall, do the benefits of using this app seem likely to outweigh inconvenience and costs to the user? Yes/No/Don’t know
21. Is there any specific clinical scenario or patient subgroup in which using the app seems particularly likely to be useful? Yes – which? ____________________________ /No/Unclear
